# Software development for modeling irregular fine protrusions formed by sputter etching

**DOI:** 10.1186/s42492-020-00047-9

**Published:** 2020-05-07

**Authors:** Sande Gao, Keijiro Nakasa, Loulin Huang

**Affiliations:** 1 Department of Mechanical Engineering, School of Science and Engineering, Meisei University, 2-1-1, Hodokubo, Hino-shi, Tokyo, 191-8506 Japan; 2 High-Tech Research Center, Hiroshima Kokusai Gakuin University, Hiroshima, 739-0321 Japan; 3 School of Engineering, Auckland University of Technology, Auckland, 1142 New Zealand

**Keywords:** Geometric modeling, Hermite surface, Fine protrusions, Software development

## Abstract

Irregular fine protrusions formed on the surface of a mechanical part through biomimetic technology can enhance the part’s properties, including tribology, self-cleaning, and light absorption. However, underlying principles for the formation of fine protrusions according to the requirements of their shapes, sizes, and material distributions have not been studied sufficiently. This paper presents the software development for modeling irregular fine protrusions, which is essential for the simulation, experimentation, and analysis of fine protrusions formed by sputter etching.

## Introduction

**Fig. 1:**
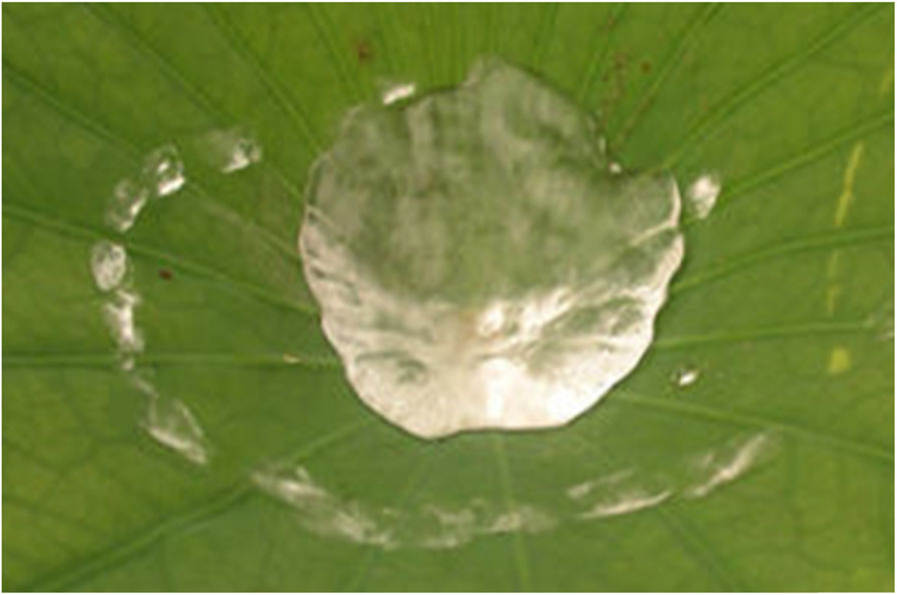
Water on the surface of a lotus leaf [2]


**Fig. 2:**
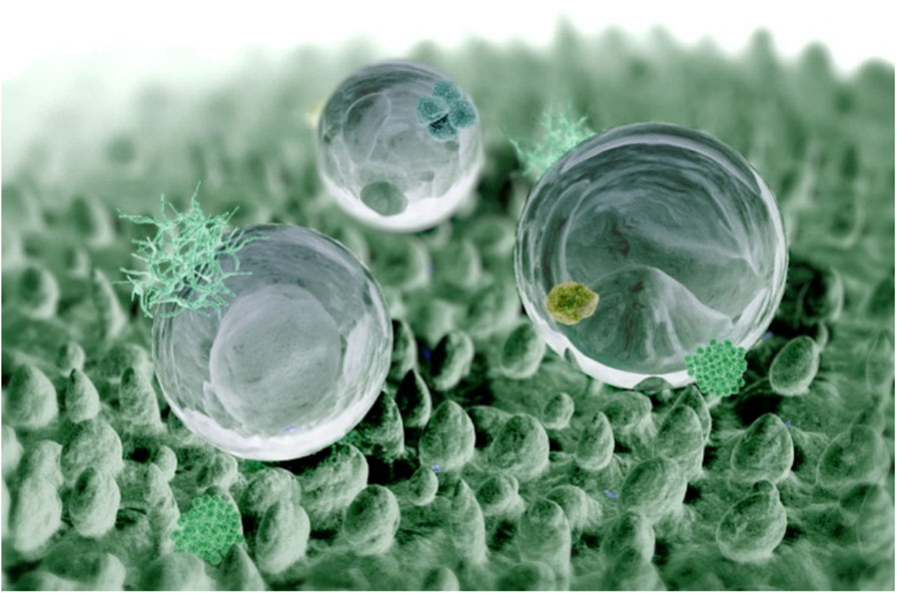
Computer graphics of a lotus leaf surface [2]

Biomimetics has resulted in new technologies inspired by biological solutions at the macro- and nano-scales found in nature, such as self-healing abilities, environmental exposure tolerance and resistance, hydrophobicity, self-assembly, and solar energy utilization [1]. An interesting example is the “lotus effect,” [2] as shown in Fig. 1. This refers to self-cleaning properties arising from ultrahydrophobicity, as exhibited by the leaves of nelumbo or lotus. Dirt particles are gathered by water droplets owing to the fine protrusions on the lotus leaf, as shown in Fig. 2, which minimizes droplet adhesion to the leaf. Superhydrophobicity has been adopted in many industrial products to prevent the drenching of cloth, paint, or concrete, as well as to deter the accumulation of rain, snow, ice, contamination, or corrosion.

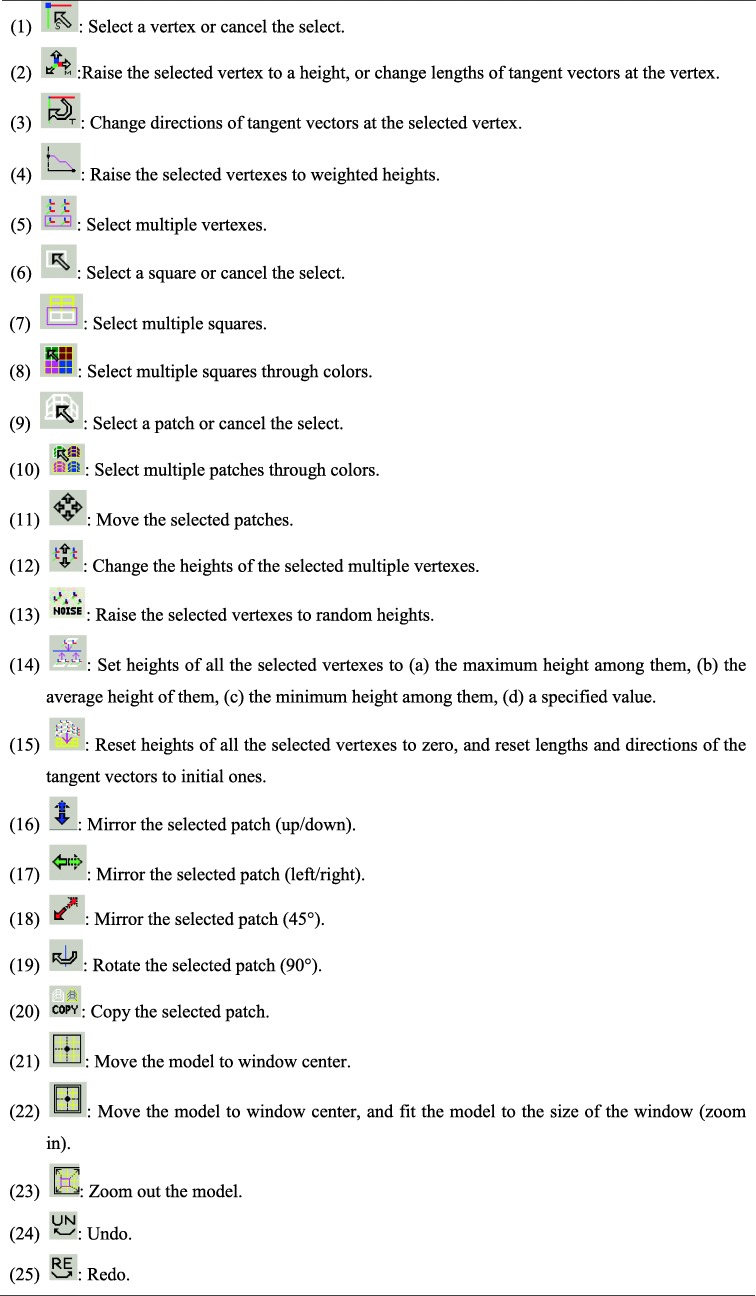

Fine protrusions on a part can be fabricated using various methods, including [3]: (1) plasma etching using a mask prepared by lithography, depositing seed materials on a substrate, or direct sputter etching without using masks; (2) chemical vapor deposition to form carbon nanotubes; (3) embedding polymers into pores of anodic alumina as a template, followed by extruding it to form nanofibers, which are then plated to form silver nanowires and gold nanorods; (4) forming nanostructures through plating and electrochemical reaction; (5) coating of nanoparticles. However, complex processes are adopted in all these methods, which is not cost effective. Moreover, in some cases the strength and heat resistance of the protrusions are insufficient for industrial applications.

**Fig. 3:**
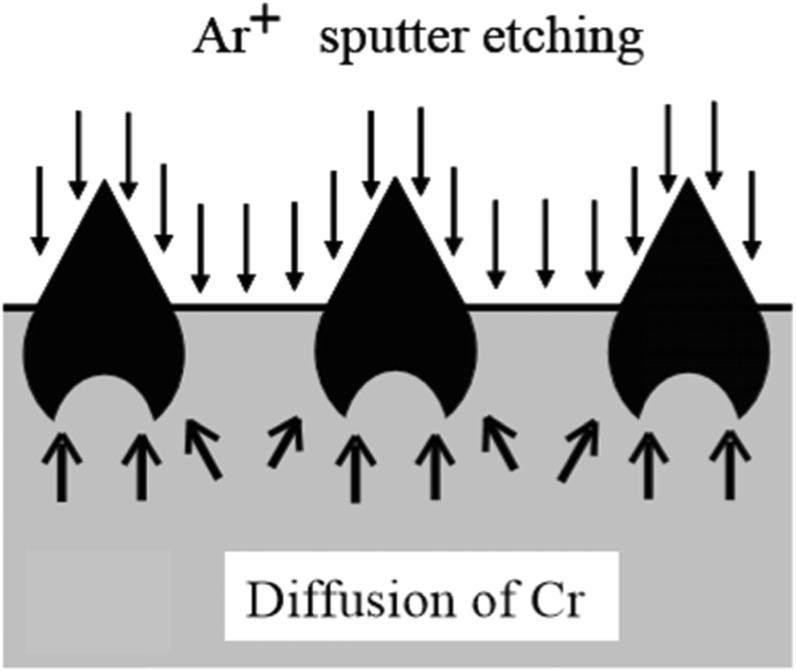
Formation mechanism of fine protrusions by sputter etching

**Fig. 4:**
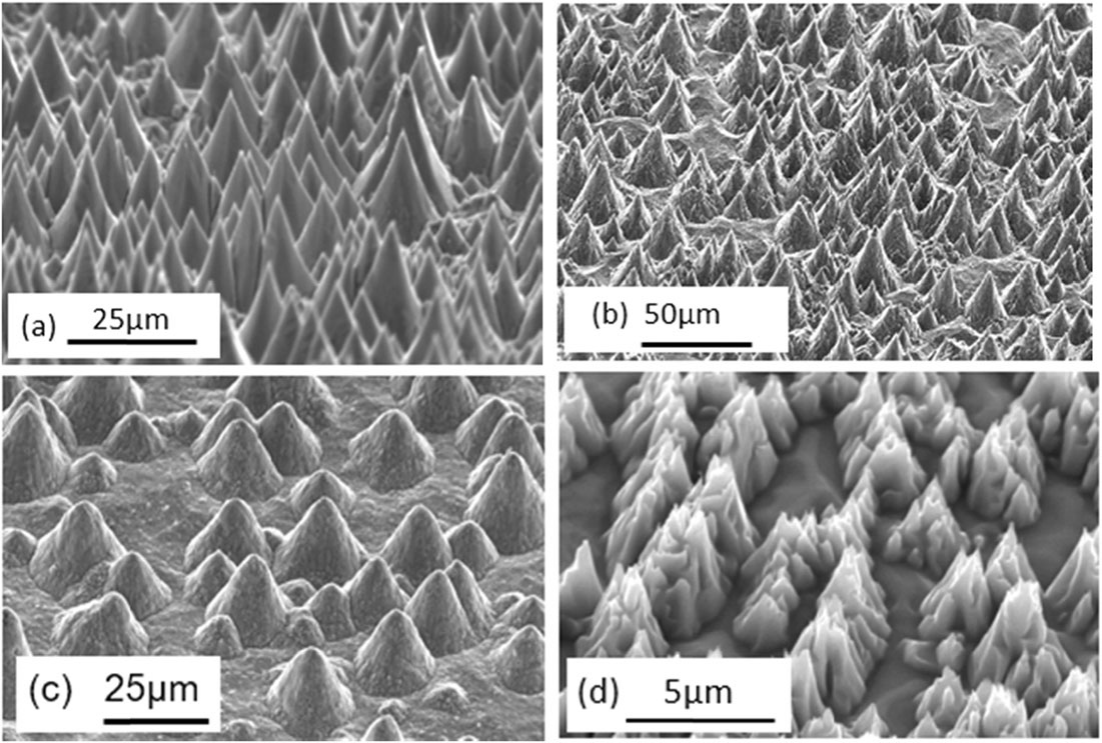
Fine protrusions formed by argon ion sputter etching

The authors discovered that fine cone-shaped protrusions can be formed on surfaces of stainless steels, low alloyed steels, or tool steels through argon ion sputter etching [4–11], a simpler and more cost effective process comparing with the above- mentioned methods. Figure 3 shows the formulation process of conical protrusions through argon ion sputter etching. The origin of the protrusion is a carbide formed on the surface. It grows to a certain size when sputter etching accelerates the diffusion of carbide-forming elements, Cr and C, from the interior of the specimen to the surface. As shown in Fig. 4, the sizes of the protrusions formed varied from 0.1 to 5 μm in diameter or width and the height/diameter ratio exceeded 1.5.

The protrusions exhibited excellent mechanical properties with only a small decrease in pitting corrosion resistance. They can be used as dies to form fine holes on a polymer film to reduce light reflections. Furthermore, they can enhance the adhesion of printing ink on a paper or cloth and improve the bonding between a transportation roll and the paper and cloth that it carries. Further, the large surface area of protrusions can be utilized as catalysts or supports of catalytic species, and the sharp tips of the protrusions can be utilized for the design of a cold emitter, temperature sensor, or heat sink of a micromachine.

**Fig. 5:**
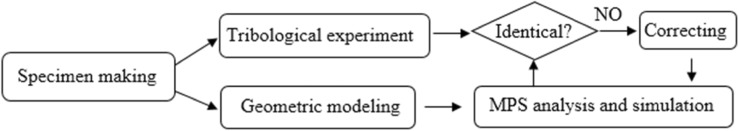
Process of investigating tribological properties of irregular fine protrusions

However, the underlying principles for the formation of fine protrusions of various shapes, sizes, and distributions have not been studied sufficiently. Hence, we conducted a study to investigate the tribological properties of irregular fine protrusions. The steps performed in the study are shown in Fig. 5. First, the irregular fine protrusions were formed on the surface of several specimens (metals and alloys) through sputter etching using argon or xenon plasma. Next, geometry models of the fine protrusions were created, and the tribological properties of the specimen when lubricant oil was applied on it were simulated and analyzed using the moving particle semi-implicit method (MPS) [12] with support from the ParticleWorks software package. The simulation and analysis results were then compared with the results obtained from the tribological experiments on the specimen. The comparison result was then used to modify the MPS model. This paper presents the software development for modeling irregular fine protrusions, an essential component of the study.

## Methods

**Fig. 6:**
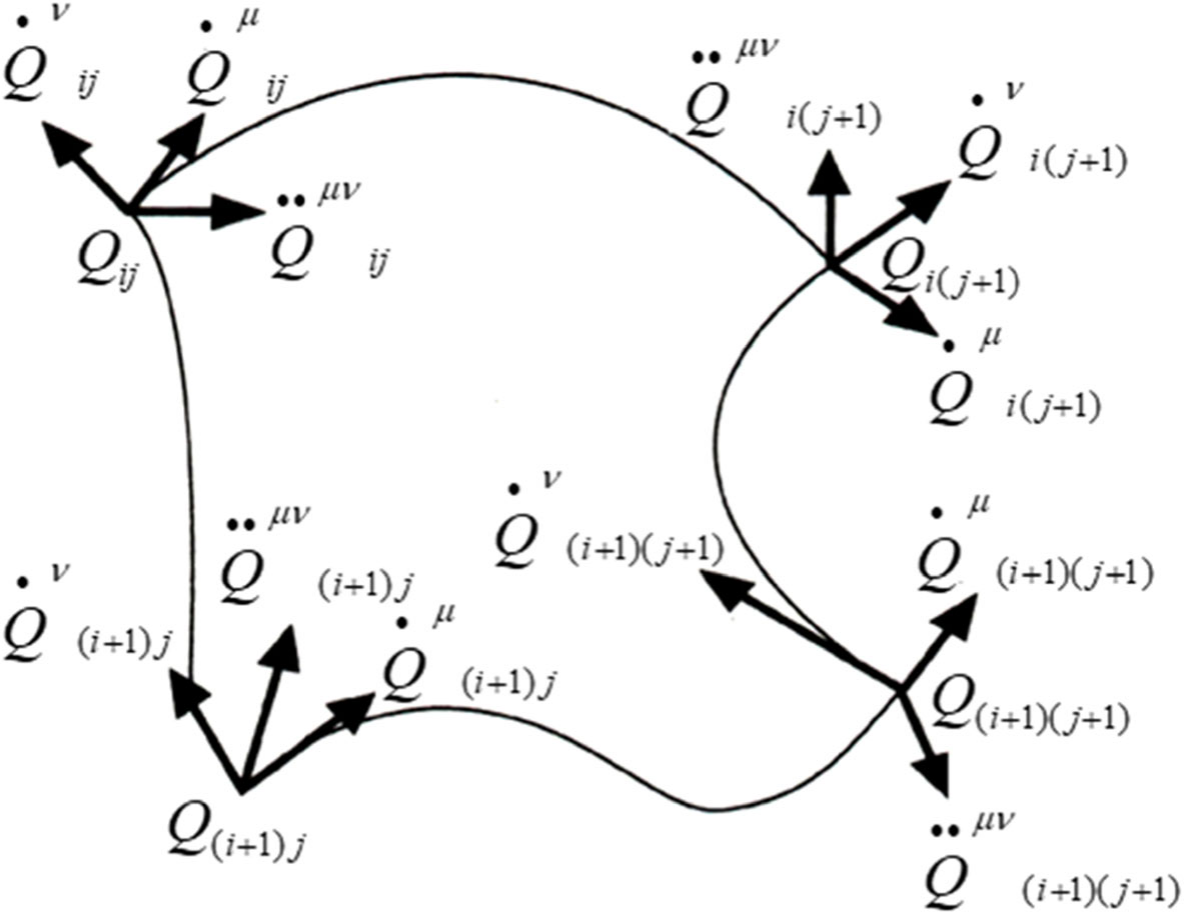
Parameters of a Hermite bicubic surface patch [13]

In this study, the geometric models of fine protrusions, in the form of a Hermite bicubic surface patch (*S*_*ij*_ at the *i*-th row and *j*-th column) derived from horizontal squares, are mathematically described by the following Hermite Eq. (1) of two parameters *u, v* (0 ≤ *u* ≤ 1, 0 ≤ *v* ≤ 1) [13, 14]. As shown in Fig. 6, a curved

1 $$ {\displaystyle \begin{array}{l}{S}_{ij}\left(u,v\right)=\left[{u}^3{u}^2u\kern0.28em 1\right]\bullet \left[\begin{array}{llll}2& -2& 1& 1\\ {}-3& 3& -2& -1\\ {}0& 0& 1& 0\\ {}1& 0& 0& 0\end{array}\right]\bullet \\ {}\left[\begin{array}{llll}{Q}_{ij}& {Q}_{i\left(j+1\right)}& {{\dot{Q}}^v}_{ij}& {{\dot{Q}}^v}_{i\left(j+1\right)}\\ {}{Q}_{\left(i+1\right)j}& {Q}_{\left(i+1\right)\left(j+1\right)}& {{\dot{Q}}^v}_{\left(i+1\right)j}& {{\dot{Q}}^v}_{\left(i+1\right)\left(j+1\right)}\\ {}{{\dot{Q}}^u}_{ij}\kern0.28em & {{\dot{Q}}^u}_{i\left(j+1\right)}& {{\ddot{Q}}^{uv}}_{ij}& {{\ddot{Q}}^{uv}}_{i\left(j+1\right)}\\ {}{{\dot{Q}}^u}_{\left(i+1\right)j}& {{\dot{Q}}^u}_{\left(i+1\right)\left(j+1\right)}& {{\ddot{Q}}^{uv}}_{\left(i+1\right)j}& {{\ddot{Q}}^{uv}}_{\left(i+1\right)\left(j+1\right)}\end{array}\right]\bullet \\ {}\left[\begin{array}{cccc}2& -3& 0& 1\\ {}-2& 3& 0& 0\\ {}1& -2& 1& 0\\ {}1& -1& 0& 0\end{array}\right]\bullet \left[{v}^3\kern0.28em {v}^2\kern0.28em v\kern0.28em 1\right]\end{array}} $$

surface patch is defined by 16 boundary conditions, including the four corner position vertexes (*Q*_*ij*_, *Q*_*i(j+1)*_, *Q*_*(i+1)j*_, *Q*_*(i+1)(j+1)*_), eight tangent vectors (${\dot{Q}}_{ij}^v$, ${\dot{Q}}_{i\;\left(j+1\right)}^v$, ${\dot{Q}}_{\left(i+1\right)j}^v$, ${\dot{Q}}_{\left(i+1\right)\left(j+1\right)}^v$, ${\dot{Q}}_{ij}^u$, ${\dot{Q}}_{i\;\left(j+1\right)}^u$, ${\dot{Q}}_{\left(i+1\right)j}^u$, ${\dot{Q}}_{\left(i+1\right)\left(j+1\right)}^u$) at the corner points (two at each point in the *u* and *v* directions), and four twist vectors (${\ddot{Q}}_{ij}^{uv}$, ${\ddot{Q}}_{i\;\left(j+1\right)}^{uv}$, ${\ddot{Q}}_{\left(i+1\right)j}^{uv}$, ${\ddot{Q}}_{\left(i+1\right)\left(j+1\right)}^{uv}$) at the corner points. The tangent vector at a corner point can be approximated by the direction and length of chord lines joining the neighboring corner points. Hence, the tangent vector information need not be input, and the calculation of the surface parameters is simplified. The software was developed with Visual C++ [15] and OpenGL [16, 17].

## Results

**Fig. 7:**
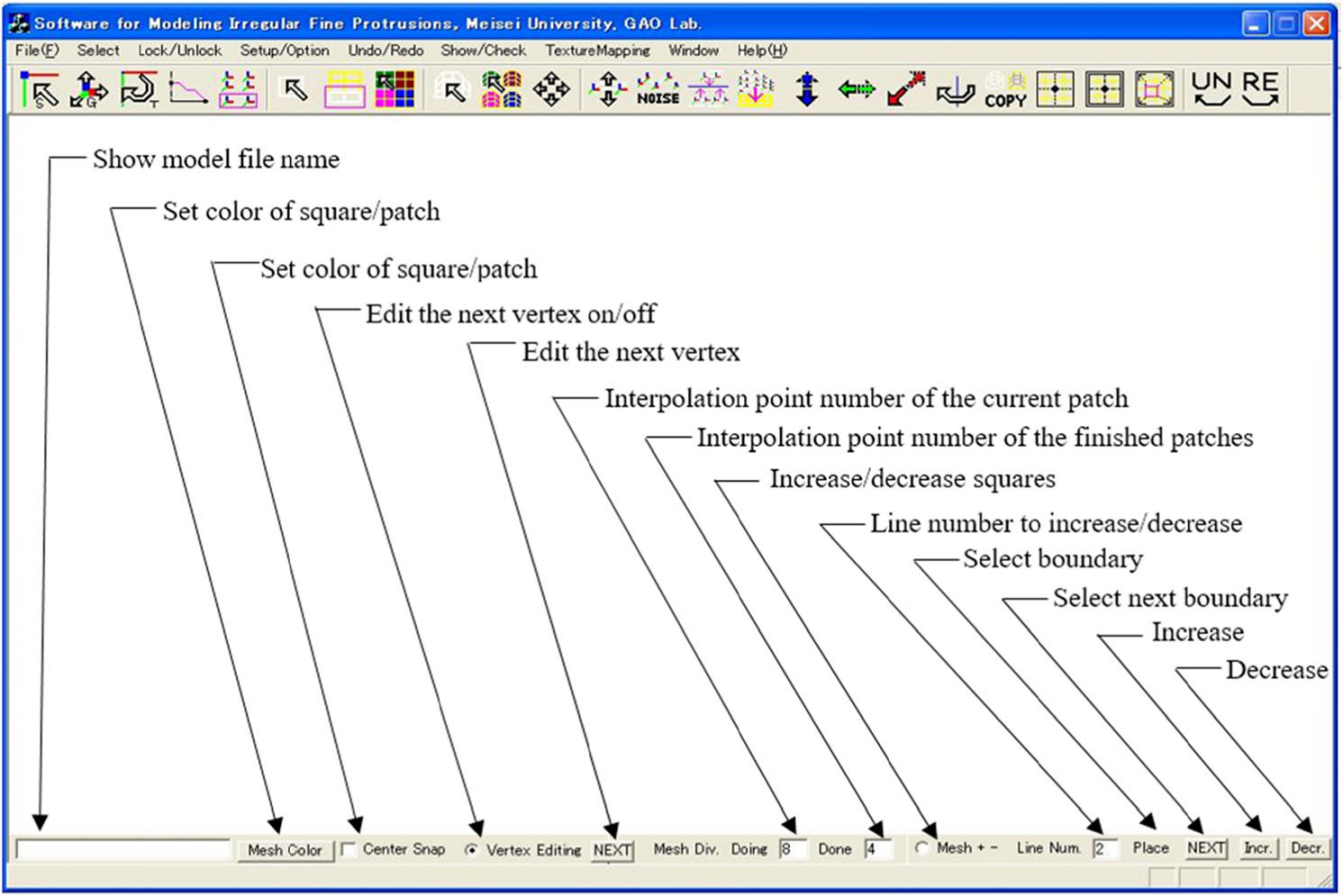
The user interface of the developed software

Figure 7 shows the user interface of the developed software. Tables 1 and 2 show the menu and toolbar.

**Table 1:** Menu

(1) File: consists of submenus “Create model”, “Open model”, “Save” and “Exit”.	
(2) Select: consists of submenus “Select all vertexes”, “Select all squares”, “Select all patches” and “Select vertexes of specified patches”.	
(3) Lock/Unlock: consists of submenus “Fix vertexes/squares/patches” and “Release vertexes/squares/patches”.	
(4) Setup/Option: consists of submenus “Set scale”, “Random variation”, “Auto-save on/off”, “Visible range”, “Set moving speed”, “Hide patches”, “Show all patches”.	
(5) Undo/Redo: consists of submenus “Go back” and “Recover”.	
(6) Show/Check: consists of submenus “Vertex information” and “Error check”.	
(7) Texture Mapping: consists of submenus “Map texture” and “Edit texture”.	
(8) Window: consists of submenus “Projection mode” and “Clean”.	
(9) Help: show information of the software.

**Table 2:**
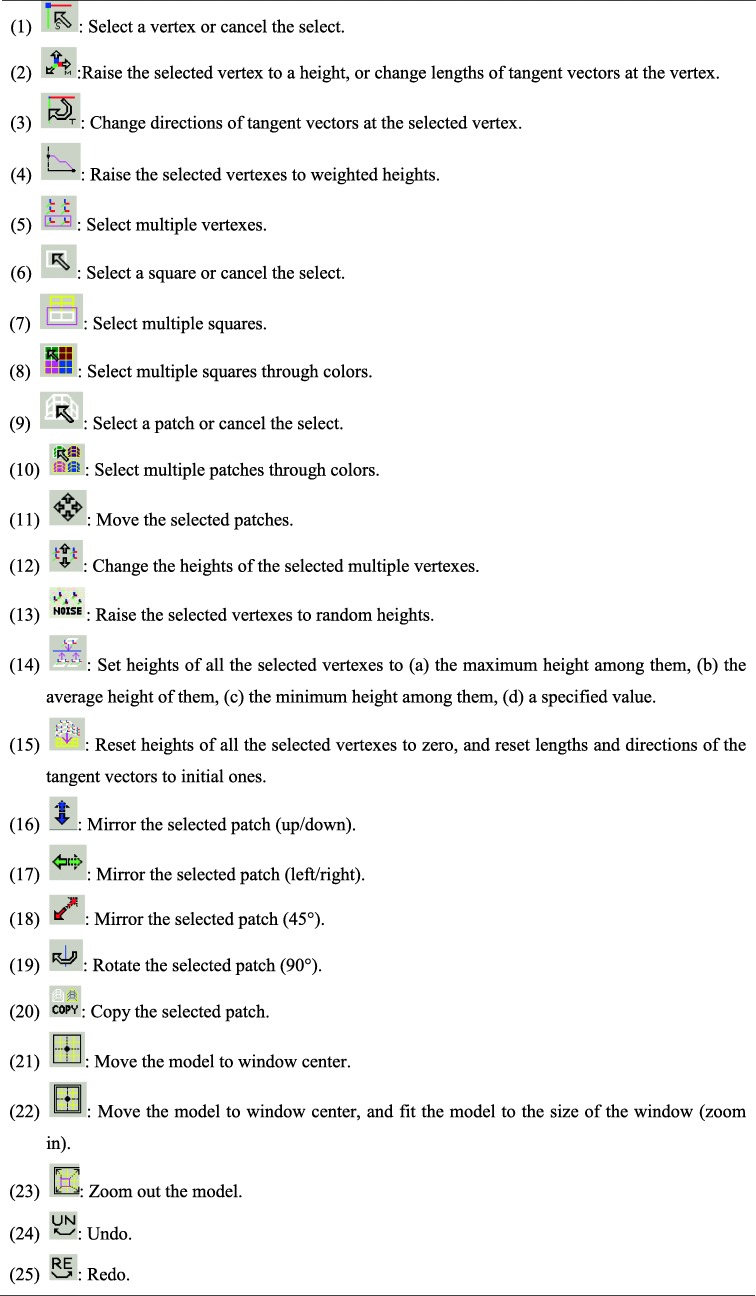
Toolbar

### Input and check boxes

The input and check boxes at the bottom of Fig. 7 are illustrated in the same figure.

### Implementation

**Fig. 8:**
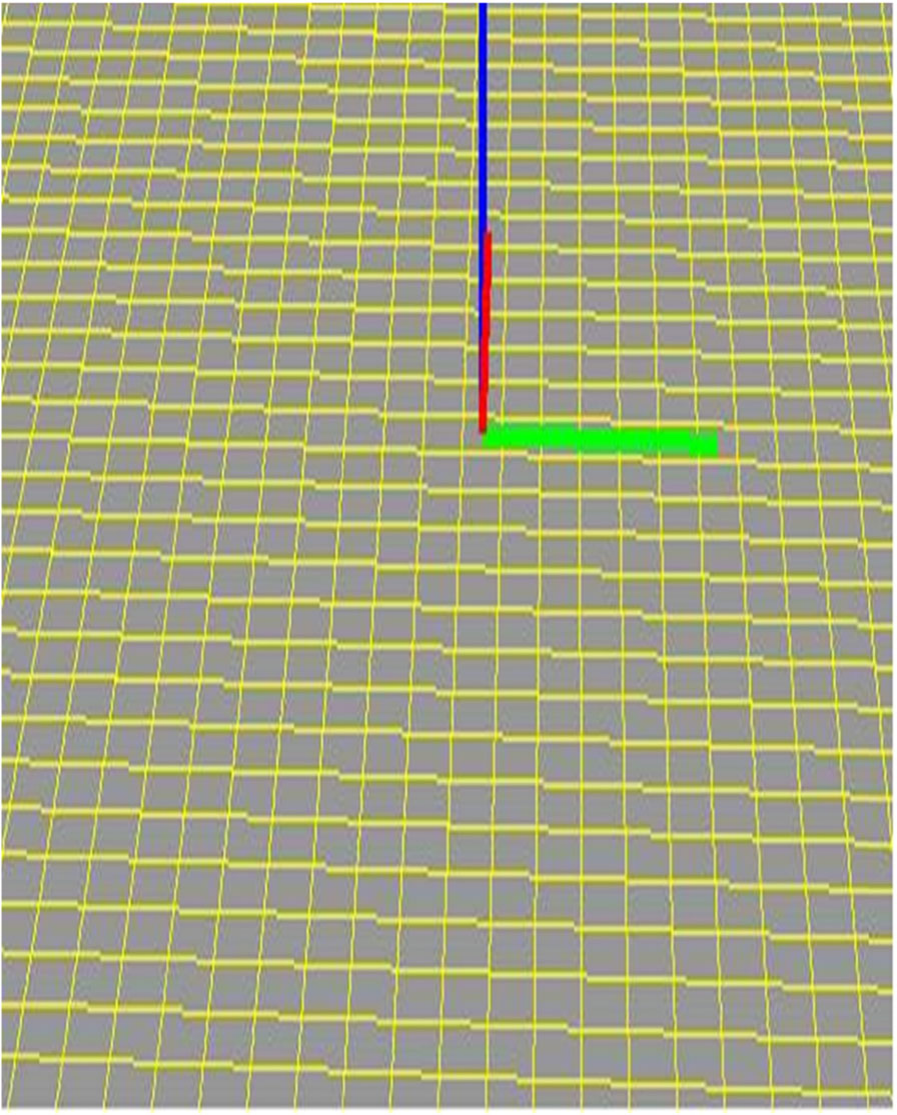
The defined squares

**Fig. 9:**
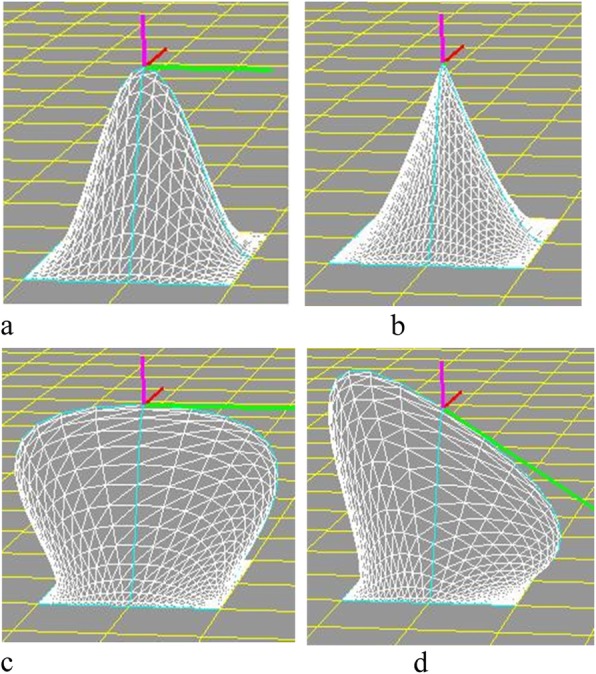
Variations in patches by changing the tangent vector length and direction. **a** Raising a vertex; **b** Shortening a vector; **c** Extending a vector; **d** Rotating a vector

**Fig. 10:**
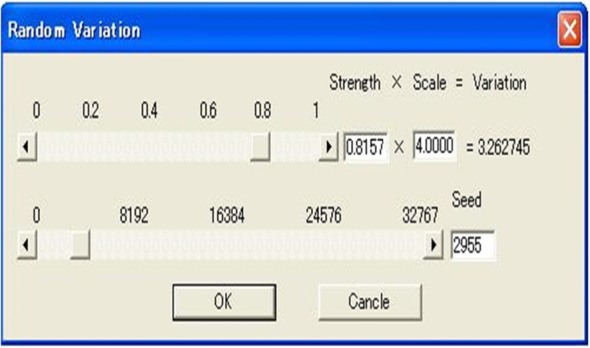
Parameters of a random shape

**Fig. 11:**
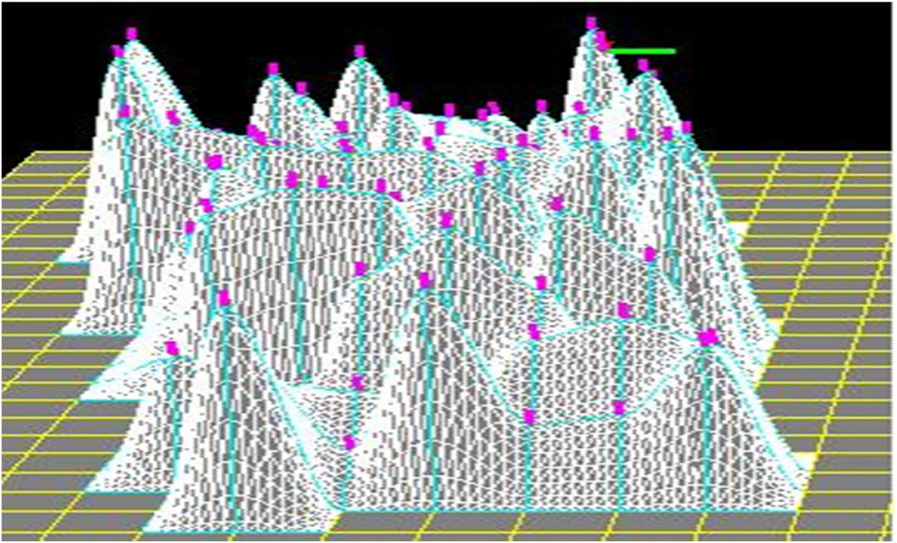
A random shape

**Fig. 12:**
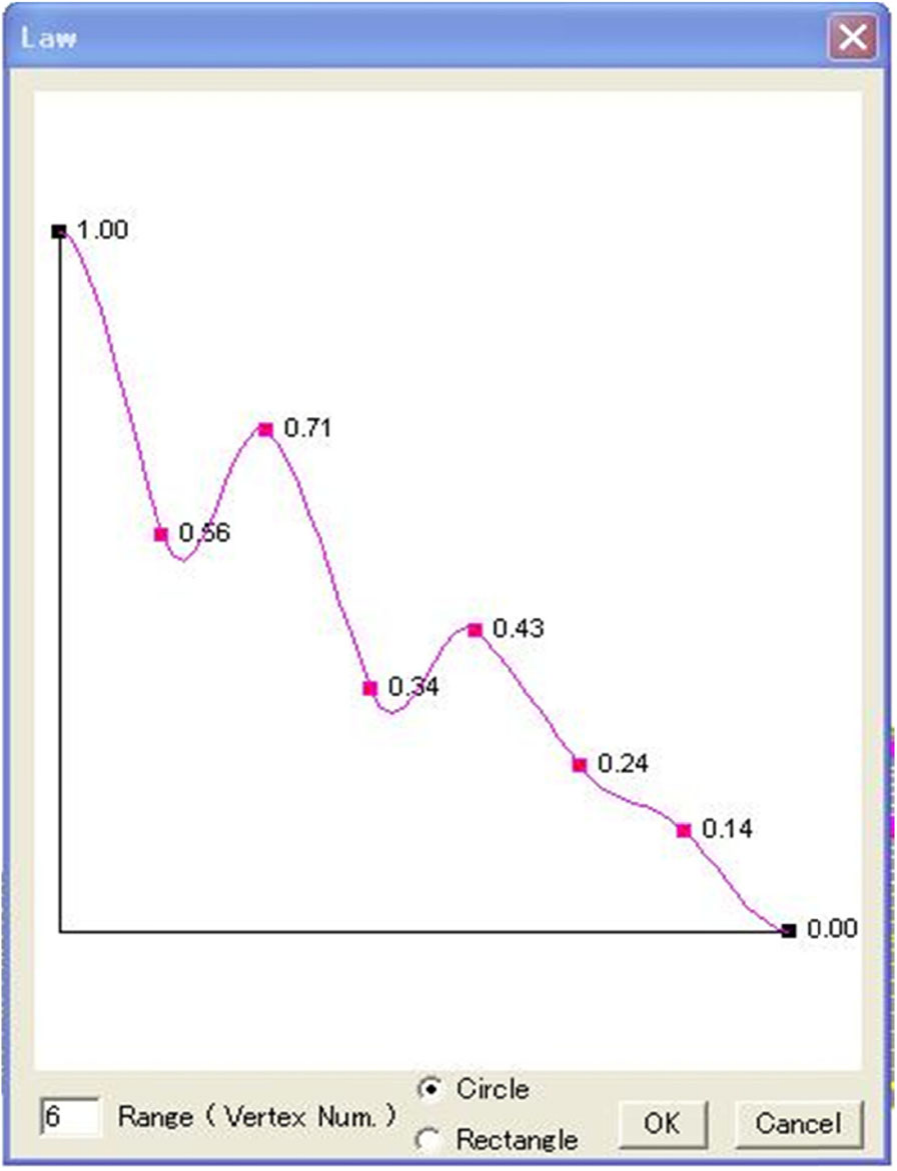
Law for a weighted shape

**Fig. 13:**
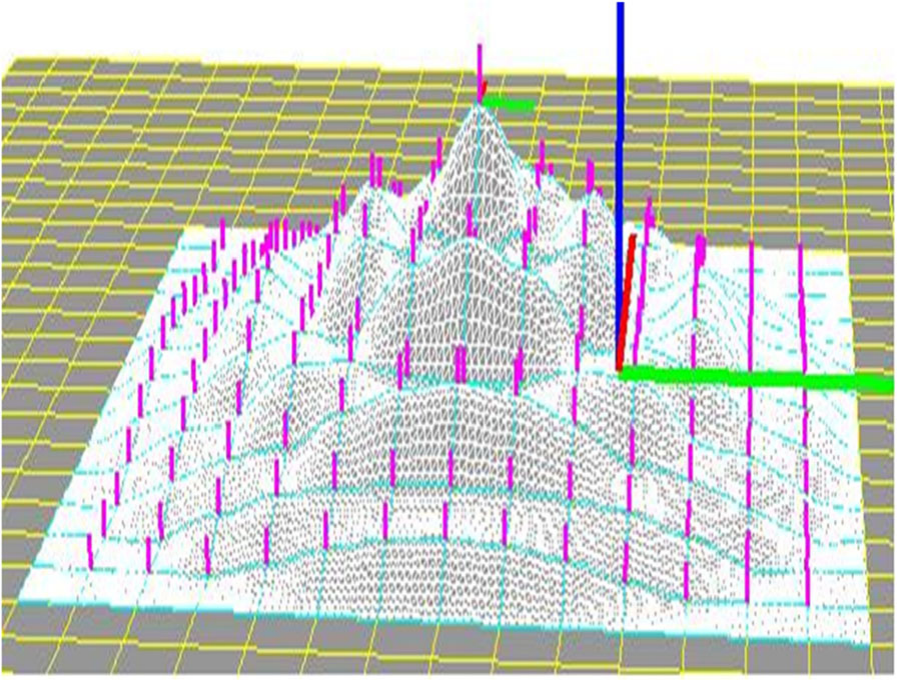
A weighted shape

**Fig. 14:**
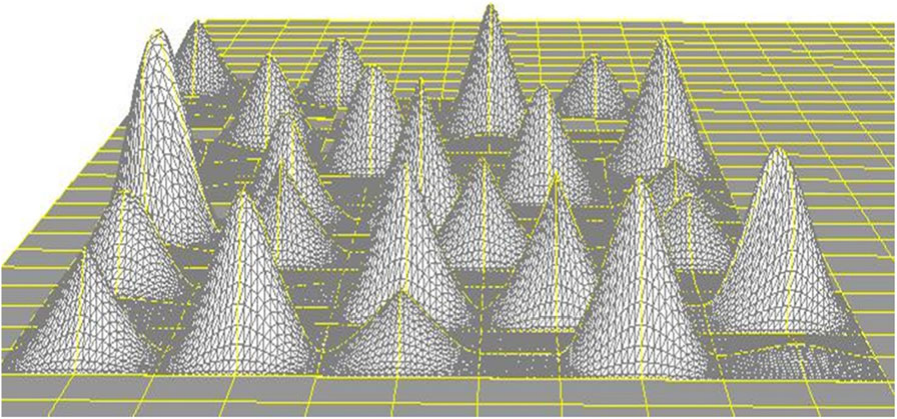
A test model of cone-shaped protrusions

The software was implemented in the following steps. First, horizontal squares were defined (Fig. 8). Next, each vertex was raised to a height to create four patches around it by computing their coordinates according to Eq. (1) to create a protrusion, and the tangent vector was shortened/extended/rotated to change the shapes of the patches with both position and tangent continuities (Fig. 9). Multiple vertexes can be raised to form protrusions with random heights effectively (Figs. 10 and 11). Furthermore, multiple vertexes can be raised to create protrusions with different heights according to the law of weights (Figs. 12 and 13). These operations were performed using a computer mouse. A test model of the cone-shaped protrusions is shown in Fig. 14. After discretizing the protrusion surfaces according to Eq. (1), the discretized points were converted into a triangular-faced mesh to create a stereolithograph (STL) file.

**Fig. 15:**
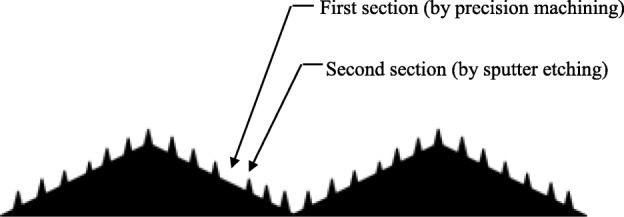
Two convex sections

**Fig. 16:**
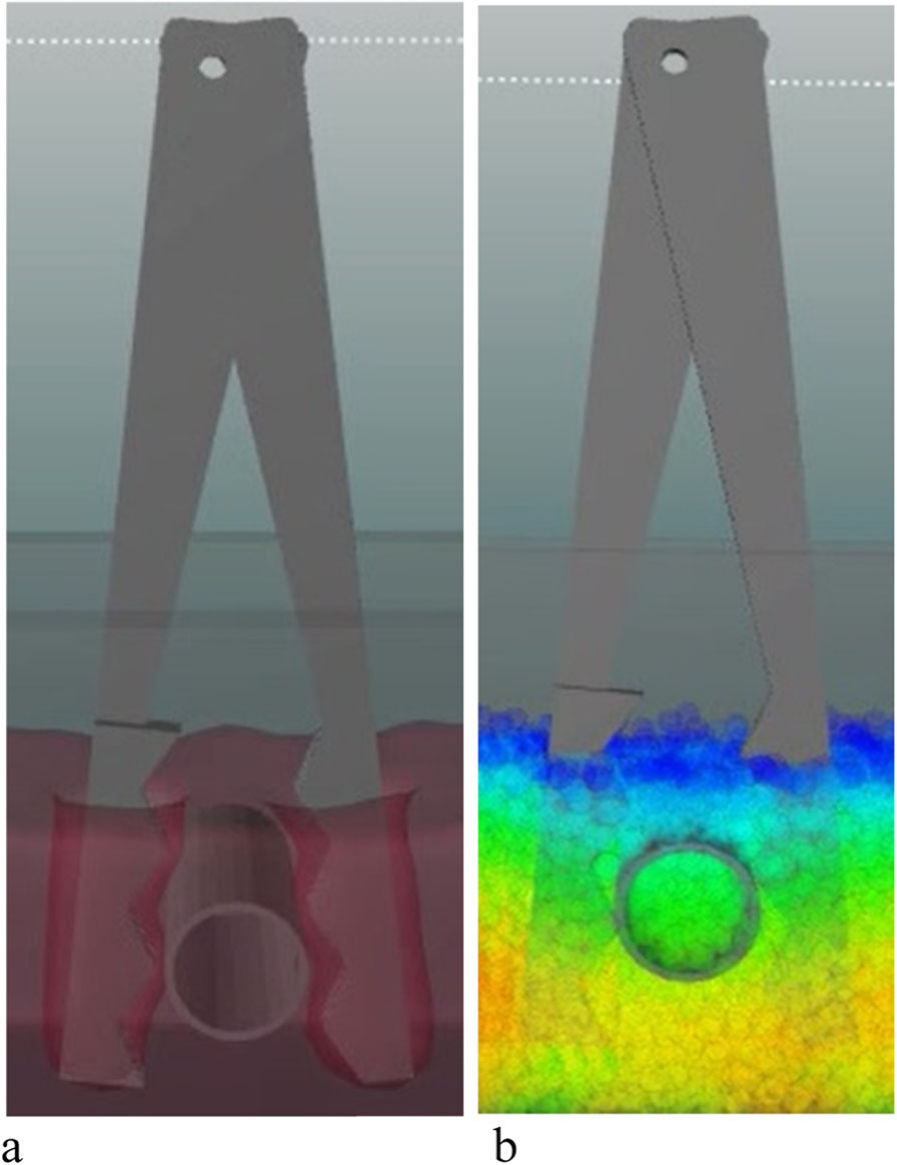
Computational simulation for developing the medical plier. **a** Model of the plier; **b** MPS simulation

This software package has been used to develop a computer simulation program for the design of a nonslip and nondestructive medical plier. As shown in Fig. 15, the working surface of the plier comprises two convex sections, in which one was produced by a high-precision machine tool in millimeters and the other was generated by sputter etching in nanometers. The second section is small and can increase friction force without damaging human body tissues. Figure 16 shows the computational simulation using the MPS software package ParticleWorks. The pressure and friction force distribution between the plier and object to be held can be observed and used to strategically assist the shape design of the two sections of the plier.

## Conclusions

A software for modeling irregular fine protrusions was developed to simulate and analyze the properties of fine protrusions formed by sputter etching. A Hermite bicubic surface was adopted in this software, although other representations such as the Bezier and B-spline surfaces can be used for free-form surfaces, and fractals can be used for some natural shapes. This was because each Hermite bicubic patch was defined with geometric conditions of four vertexes, which rendered it easy to describe local variations of a shape. By contrast, each Bezier surface or B-spline surface was defined with 16 vertexes, which was suitable for defining large smooth surfaces but not ideal for modeling irregular fine protrusions. Because the statistical character of each fine protrusion differed from that of the entire surface, fractals were not applicable.

Using this software, fine protrusions in various shapes, such as cone, ring, sphere, and pipe can be created. The maximum number of modeled fine protrusions was approximately 10^6^, which was sufficient for the MPS analysis and simulation. A protrusion can be created rapidly by clicking on a computer mouse. Large-scale of protrusions can be created effectively with the random height method. The STL data of the created protrusions can be input to and processed by the MPS software package ParticleWorks. Furthermore, the software developed is applicable to geographic information systems. Its open structure allows the functions to be enhanced to improve the efficiency and accuracy of modelling.

## Data Availability

Not applicable.

## Abbreviations

MPS: Moving particle semi-implicit
STL: Stereolithograph
